# Epstein-Barr virus reactivation is not causative for post-COVID-19-syndrome in individuals with asymptomatic or mild SARS-CoV-2 disease course

**DOI:** 10.1186/s12879-023-08820-w

**Published:** 2023-11-15

**Authors:** Alexandra Domnica Hoeggerl, Verena Nunhofer, Wanda Lauth, Natalie Badstuber, Nina Held, Georg Zimmermann, Christoph Grabmer, Lisa Weidner, Christof Jungbauer, Nadja Lindlbauer, Heidrun Neureiter, Tuulia Ortner, Maria Flamm, Jürgen Osterbrink, Eva Rohde, Sandra Laner-Plamberger

**Affiliations:** 1 Department of Transfusion Medicine, University Hospital of Salzburg (SALK), Paracelsus Medical University (PMU) Salzburg, Müllner-Hauptstraße 48, Salzburg, 5020 Austria; 2 Team Biostatistics and Big Medical Data, IDA Lab Salzburg, PMU Salzburg, Strubergasse 16, Salzburg, 5020 Austria; 3 Research and Innovation Management, PMU Salzburg, Strubergasse 16, Salzburg, 5020 Austria; 4 Department of Psychological Assessment, Institute of Psychology, Paris-Lodron-University of Salzburg, Salzburg, Austria; 5 Austrian Red Cross, Blood Service for Vienna, Lower Austria and Burgenland, Wiedner Hauptstraße 32, Vienna, 1040 Austria; 6 Institute of General Practice, Family Medicine and Preventive Medicine, PMU Salzburg, Strubergasse 21, Salzburg, 5020 Austria; 7 Institute of Nursing Science and Practice, PMU Salzburg, Strubergasse 21, Salzburg, 5020 Austria; 8 Spinal Cord Injury and Tissue Regeneration Centre Salzburg, PMU Salzburg, Strubergasse 21, Salzburg, 5020 Austria

**Keywords:** SARS-CoV-2, COVID-19, Post-COVID-19-syndrome (PCS), Long-COVID, EBV

## Abstract

**Purpose:**

Post-COVID-19-Syndrome (PCS) frequently occurs after an infection with severe acute respiratory syndrome coronavirus-2 (SARS-CoV-2). However, the understanding of causative mechanisms is still limited. Aim of this study was to determine the PCS rate among SARS-CoV-2 seropositive blood donors as representatives of supposedly healthy adults, who had experienced an asymptomatic or mild COVID-19 disease course, and to examine whether Epstein-Barr virus (EBV) is reactivated in individuals reporting PCS.

**Methods:**

The PCS rate was determined using questionnaires that included questions about infection and persistent symptoms. Pre-pandemic blood samples and samples collected at regular, pre-defined times after a SARS-CoV-2 infection were analysed for neopterin, a marker for antiviral immune responses, by an enzyme-linked immunosorbent assay (ELISA). Additionally, we determined the rate of SARS-CoV-2 anti-N total antibodies using an electrochemiluminescence immunoassay (ECLIA). Furthermore, quantitative real-time polymerase chain reaction (qPCR) to detect EBV DNA and ECLIA screening for EBV viral capsid-antigen (VCA) IgM, IgG and EBV nuclear antigen 1 (EBNA) IgG were performed.

**Results:**

Our data reveal that 18% of all infections result in PCS, with symptoms lasting for up to one year. In individuals reporting PCS, no elevated levels of neopterin were detected, indicating no persisting pro-inflammatory, antiviral immune response. SARS-CoV-2 antibody levels were declining in all participants in comparable manner over time, pointing to a successful virus clearance. In individuals with PCS, no EBV DNA could be detected. Furthermore, no differences in EBV specific antibody levels could be shown in PCS groups compared to non-PCS groups.

**Conclusion:**

Our data suggest that PCS in per se healthy, immunocompetent adults cannot be ascribed to a reactivation of EBV.

**Supplementary Information:**

The online version contains supplementary material available at 10.1186/s12879-023-08820-w.

## Introduction

Since the beginning of the pandemic in 2020, infections with severe acute respiratory syndrome coronavirus-2 (SARS-CoV-2) are challenging healthcare, social and economic systems worldwide. The care of many acute patients suffering from coronavirus disease-2019 (COVID-19) who frequently required intensive care including external ventilation was a major challenge. New virus variants emerged quickly, still causing repeated infections worldwide. Furthermore, infections with SARS-CoV-2 have led to late and long-lasting health impairment, which is termed post-COVID-19-syndrome (PCS), post-acute sequelae of COVID-19 (PASC) or long-COVID. In this study, the terminology PCS will be used henceforth.

The World Health Organisation (WHO) defined PCS as a condition that usually occurs three months from the onset of COVID-19 with symptoms that last for at least two months and cannot be explained by an alternative diagnosis [1]. The prevalence of PCS is estimated to range between 5% and 70% [2–5]. This broad range may be ascribed to different study designs and recruitment strategies, but may also be related to the causative virus variant: higher PCS rates were described for individuals infected with the wild type compared to the omicron variant [6–8]. Furthermore, it is suggested that vaccination confers partial protection [9]. Currently it cannot be predicted precisely who is at risk of developing PCS. PCS is reported for patients hospitalized due to a severe disease course but also for individuals presenting rather mild symptoms or even an asymptomatic course of the SARS-CoV-2 infection [10, 11]. PCS symptoms cover a very broad spectrum: more than 200 different symptoms, ranging from respiratory symptoms, pain affecting bones, muscles and joints, psycho-cognitive impairments such as anxiety and depression to physical fatigue-associated symptoms, have been described so far [11–14]. Due to the large numbers of reported cases, it is obvious that PCS creates a considerable amount of chronic patients, causing burden to the individuals affected, but also to economy and health systems worldwide. Therefore, to date a lot of effort is invested aiming to identify the causative mechanisms for this health issue.

As reviewed recently, the persistence of SARS-CoV-2 in different organs could be one of the pathogenic mechanisms driving the development of PCS [15]. Activated autoimmune responses and other persistent and uncontrolled inflammatory processes, including sustained presence of pro-inflammatory cells, altered cytokine production, hampered virus recognition and clearance mechanisms, are also suggested to be pivotal for developing PCS [16, 17]. The reactivation and the response to unrelated viruses such as Epstein-Barr virus (EBV) is discussed as another causative factor of PCS. EBV, a double stranded DNA virus of the herpesvirus family, is one of the most common viruses in humans with more than 90% of adults worldwide showing evidence for a previous infection [18]. Two studies have suggested a direct correlation between the reactivation of EBV and the severity of COVID-19 disease course [19, 20]. A few data also indicate that EBV could be reactivated in individuals suffering from PCS [21, 22].

Aim of this study was to investigate a putative EBV reactivation in supposedly healthy adults after asymptomatic or rather mild COVID-19 disease course without hospitalisation that are admitted to blood donation and report PCS. Using online surveys, we determined the rate of PCS among seropositive participants and documented the symptoms experienced. We next compared the amount of neopterin, an unspecific prognostic marker for pro-inflammatory, active antiviral immune responses [23], between individuals with and without PCS at different points in time. To ensure that a SARS-CoV-2 infection had been experienced and to examine the developmental course of specific antibodies over time, SARS-CoV-2 anti-N total antibodies were monitored, which are produced after an infection but not after vaccination. Furthermore, we screened for the presence of EBV DNA and examined the levels of EBV viral capsid-antigen (VCA) IgM, VCA IgG and EBV nuclear antigen 1 (EBNA) IgG antibodies.

## Materials and methods

### Ethical statement

For this study, human residual serum utilized for routine laboratory diagnostics as part of standard blood donation processing according to European and local regulations was used. All blood donors gave signed informed consent on the use of leftover material for research purposes. Blood donors seropositive for SARS-CoV-2 were invited to participate in our study. After signing informed consent, further blood samples were collected at regular, pre-defined points in time. The ethical committees of the Federal State of Salzburg, Austria and the Paracelsus Medical University Salzburg approved the study (ethical vote numbers 1004/2021 and SS22-0026-0026). The work described was carried out in accordance with the 1964 Helsinki Declaration and its later amendments or comparable ethical standards. Samples were processed anonymously to protect privacy of each donor.

### Study design

**Fig. 1:**
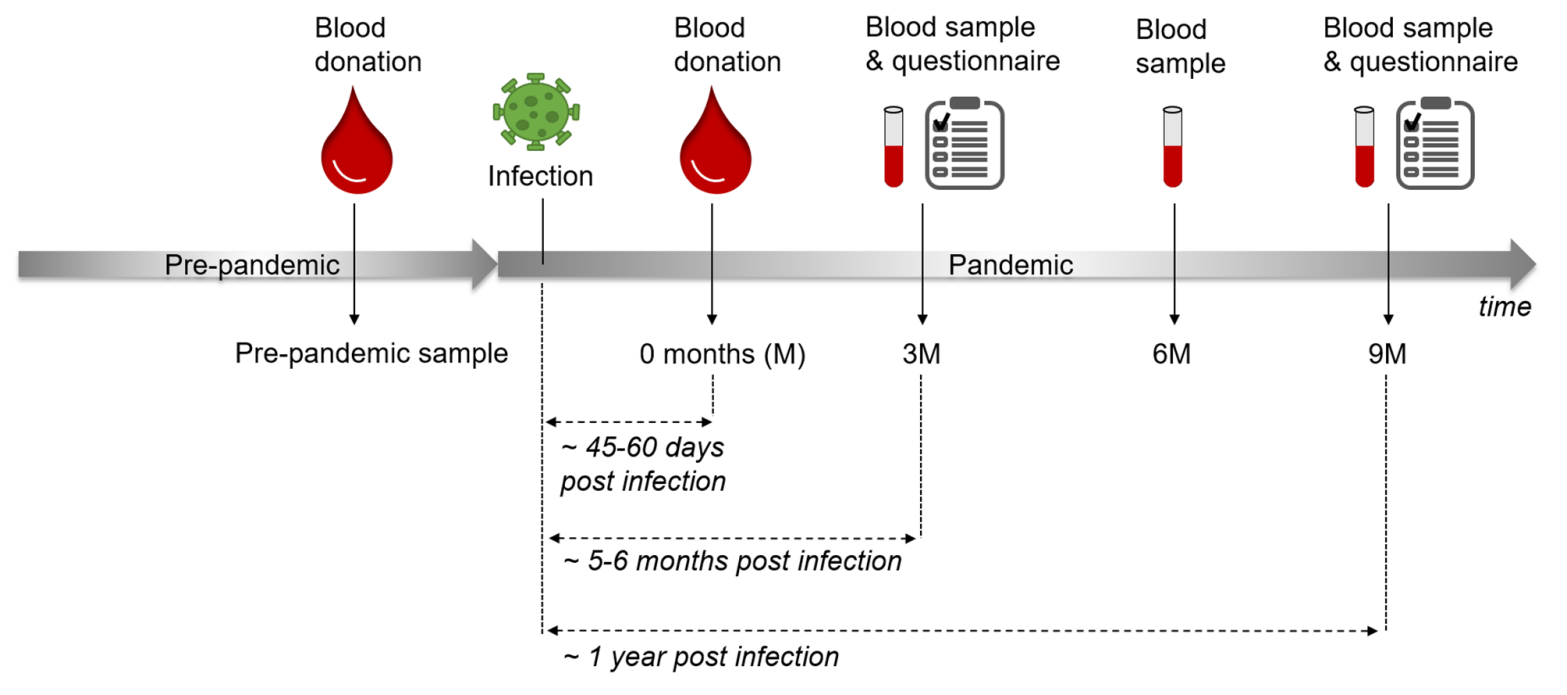
Several different samples of each study participant were examined: (1) pre-pandemic serum/plasma sample, which was collected in the course of a voluntary blood donation in the pre-pandemic period in 2019. (2) Serum/plasma of the blood donation, which was voluntarily done on average 45–60 days after a SARS-CoV-2 infection (0 months (0 M) sample). (3) Samples, which were collected 3 (~ 5–6 months post infection), 6 and 9 months (~ 1 year post infection) after the seropositive blood donation. In addition, 3 and 9 months after the seropositive blood donation, a questionnaire regarding SARS-CoV-2 infection and persisting symptoms was to be filled in. M = months

As described in our previous studies [24, 25], all blood donors had a brief health screening and completed a written questionnaire including an informed consent on pathogen screening prior to blood donation. Blood donors tested serologically positive for SARS-CoV-2 anti-N total antibodies were invited to take part in the present study, providing negative screening for other infectious disease parameters tested (serological and molecular biological screening for HIV, HBV, HCV, HAV, parvovirus B19, Treponema pallidum and WNV as a part of the standard screening of each blood donation). No further preselection was done. In total, 400 seropositive blood donors willing to participate in this longitudinal study were included after signing informed consent between December 2020 and September 2022. As depicted in Fig. 1, several serum and plasma samples of each individual were analysed at different points in time. Besides the samples from the initial SARS-CoV-2 seropositive blood donation (0 months), which induced the participation in our study, we examined further samples collected 3, 6 and 9 months after the initial blood donation. In addition, we also used serum and plasma samples of the participants collected in the course of a voluntary blood donation in the pre-pandemic time between January and September 2019. According to blood donation regulations, a retention samples of each blood donation must be conserved for 2 years. After the legal retention period had expired, retained samples from study participants were used as pre-pandemic samples.

### Online surveys

Study participants were invited to answer two online questionnaires regarding the time and course of the SARS-CoV-2 infection, symptoms during the infection, persisting symptoms and other health-associated issues such as general health status and known comorbidities. The online questionnaires were to be filled in 3 and 9 months after the initial blood donation (Fig. 1). Regarding persisting symptoms, participants were asked about symptoms they personally attributed to the SARS-CoV-2 infection without involving the terminology “long-COVID” or “PCS”. No list of choices was offered, but participants named symptoms. Participants categorized into the PCS groups fulfill the definition of PCS according to the WHO with symptoms occurring three months from the onset of COVID-19 with symptoms and that last for at least two months [1]. Lime Survey (https://www.limesurvey.org, last accessed 13.05.2023) was applied to administer the questionnaires.

### Serological screening for neopterin

To determine the level of the inflammatory marker neopterin prior and after a SARS-CoV-2 infection, we applied the quantitative neopterin enzyme-linked immuno-sorbent assay (ELISA) (IBL International, Hamburg, Germany) according to manufacturer’s instructions applying an ETI-MAX 3000 fully automated microtiter plate analyser (DiaSorin, Saluggia, Italy). Results are given as nmol/L, with all values > 11 nmol/L being considered as elevated and thus indicate enhanced pro-inflammatory, anti-viral immune processes.

### Serological screening assays for SARS-CoV-2 and EBV antibodies

As already described in our previous studies [24, 25], the Elecsys Anti-SARS-CoV-2 (ACOV2) total antibody electrochemiluminescence immunoassay (ECLIA, Roche Diagnostics, Basel, Switzerland) was applied to screen for SARS-CoV-2 anti-N total antibody (including IgM, IgG and IgA) using a cobas8000-e801 device (Roche Diagnostics) according to manufacturer’s instructions. In this semi-quantitative test, a recombinant protein of the viral nucleocapsid (N) antigen is used to determine antibodies against SARS-CoV-2. The results of this screening approach are based on the sample signal to cut-off ratio with values < 1.0 corresponding to negative results and values ≥ 1.0 corresponding to positive results. According to the manufacturer, this screening assay is able to detect but not discriminate all SARS-CoV-2 variants known so far.

For this study, we applied three different Elecsys EBV antibody screening assays (all Roche Diagnostics) on the cobas8000-e801 device (Roche Diagnostics) according to manufacturer’s instructions: Elecsys EBV VCA IgM, Elecsys EBV VCA IgG and Elecsys EBV EBNA IgG. EBV VCA IgM is an early serological marker of an EBV infection and can be detected 3–6 months post infection and reactivation [26–28]. Like EBV IgM, VCA IgG is produced at an early stage of EBV infection, typically at clinical onset of disease, but in contrast, it is detectable for lifetime. EBNA1 IgG is produced during later stages of EBV infection (usually 6–12 weeks post infection) and is detectable for the rest of life [26, 29]. As already described for the Elecsys Anti-SARS-CoV-2 assay, the results of these semi-quantitative EBV-specific antibody screening approaches are based on the sample signal to cut-off ratio, with values < 1.0 corresponding to negative results and values ≥ 1.0 corresponding to positive results.

### Nucleic acid testing (NAT) based on real-time polymerase chain reaction (qPCR)

For the detection and quantitative determination of EBV DNA in EDTA plasma from study participants, the cobas EBV nucleic acid amplification test (Roche Diagnostics) was applied using a fully automated cobas 6800 molecular analyser (Roche Diagnostics) according to manufacturer’s instructions. In brief, the plasma sample was amplified together with a non-EBV DNA quantification standard (QS) applying TaqMan Polymerase and specific TaqMan probes. The use of the QS in combination with three external controls (a high titre positive control, a low titre positive control, and a negative control) allowed the quantitative determination of the viral load and monitoring of the entire sample preparation and PCR amplification process.

### Statistical analysis

For data analysis, in order to relate to antibody and neopterin levels found in pre-pandemic blood donation samples, the relative change from baseline (i.e., pre-pandemic blood donation samples) was calculated for blood samples collected at points in time 0 M and 3 M. For descriptive analysis of data, mean, standard deviation, median and interquartile range were calculated. Boxplots were used for visualization. Due to the unbalanced sample size per group, individuals were categorized into three groups as follows: Individuals from group 1 were considered asymptomatic, individuals from group 2 were considered symptomatic without PCS, and individuals from groups 3, 4, and 5 were considered symptomatic with PCS. Statistical analysis (hypothesis testing) was done only for these three groups. Nonparametric ANOVA tests for repeated measures were performed to compare the interactions between group and time [30]. Additionally, to compare differences between groups at individual points in time, Kruskal-Wallis tests were applied and to detect differences in the groups regarding nominal variables (e.g. symptoms) Fisher’s exact test was calculated. Bonferroni-Holm was used to adjust for multiplicity. For all statistical tests the two-sided significance level α = 0.05 was assumed. All analyses were carried out using the statistical software package R [31].

### Graphic software used for illustrations

**Fig. 2:**
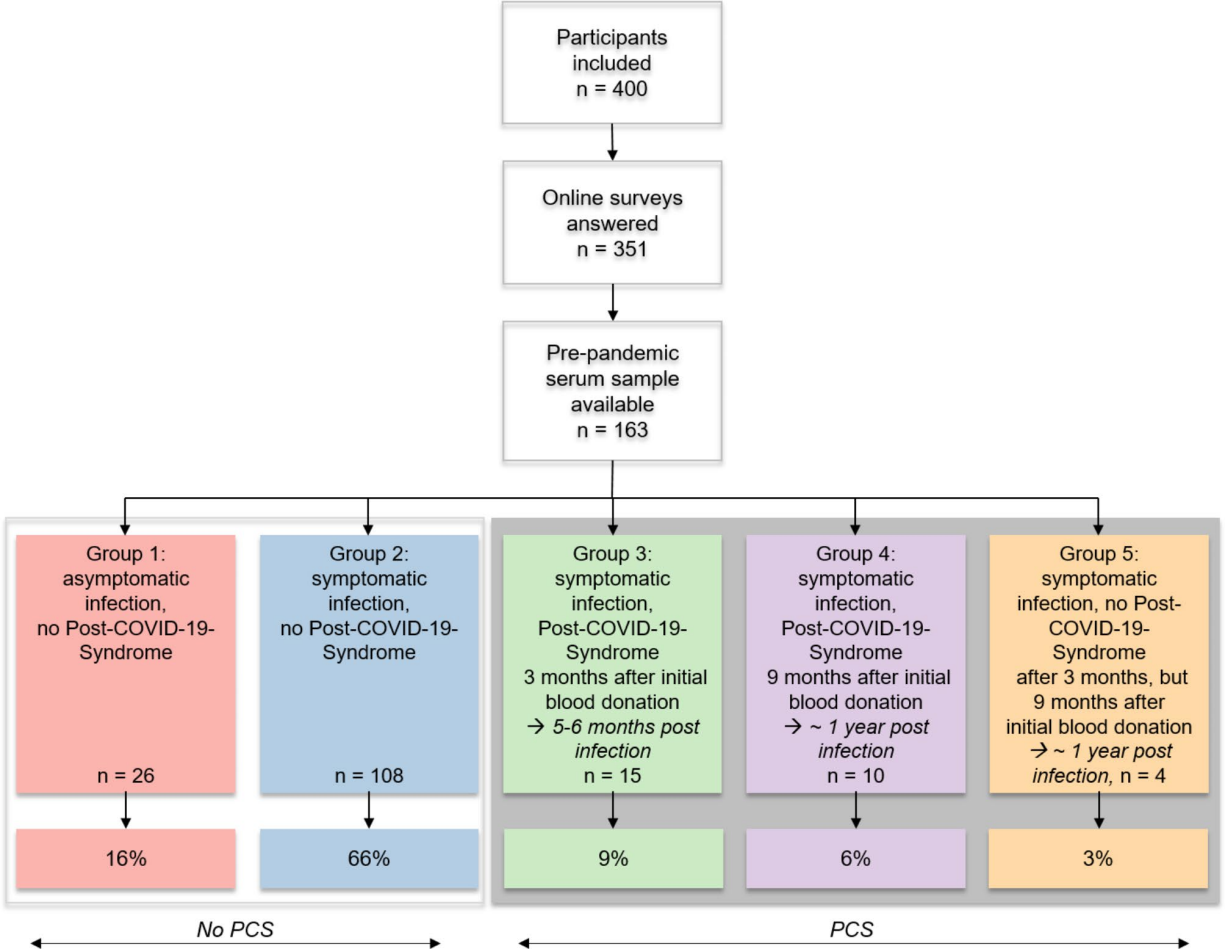
Four hundred participants were included in our study with 351 answering the online surveys 3 and 9 months after the initial seropositive blood donation. For 163 individuals samples were available from the pre-pandemic period in 2019 due to the legal sample retention requirements of blood donations. These individuals were grouped according to the course of COVID-19 and putative PCS-symptoms into five subgroups as indicated. BD = blood donation

**Fig. 3:**
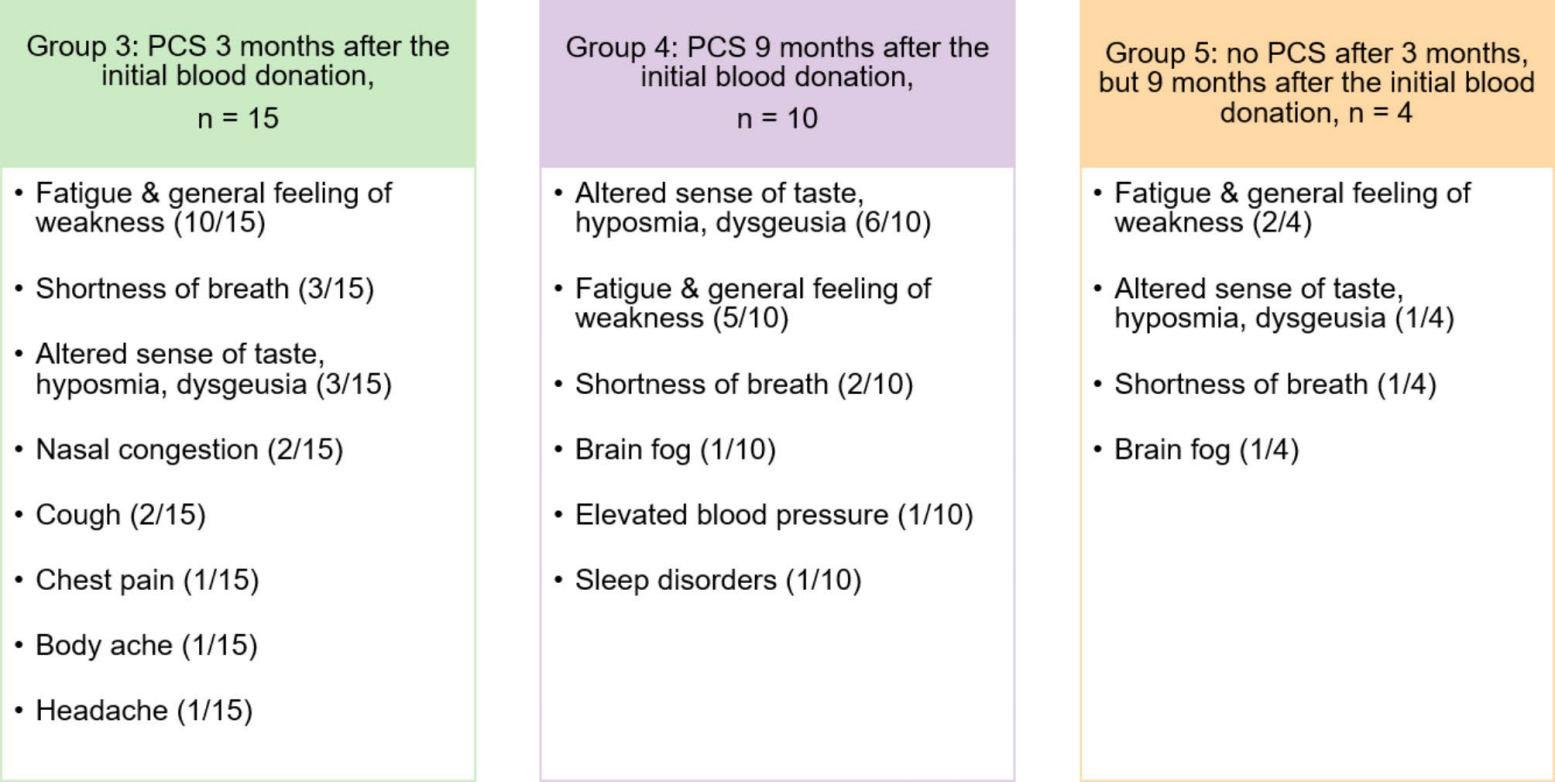
The three groups of participants reporting PCS for different time spans after a SARS-CoV-2 infection share fatigue, an altered sense of taste (including hyposmia and dysgeusia) and shortness of breath as the three most common symptoms. Symptoms reported are listed in the corresponding box for each group with the number of individuals reporting a particular symptom given in brackets

**Fig. 4:**
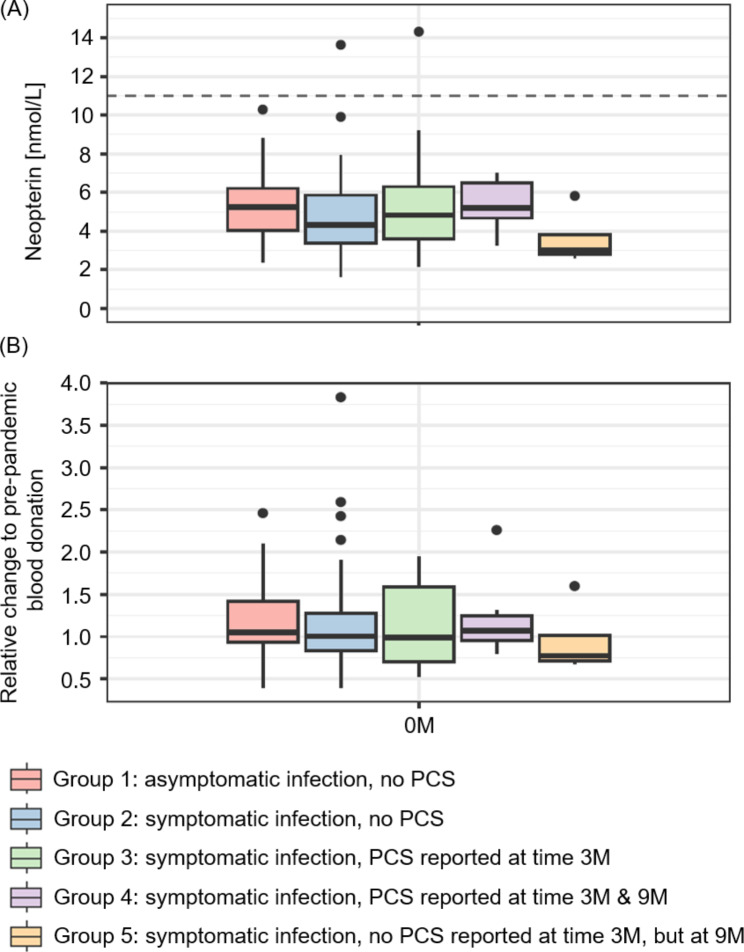
Neopterin levels in individuals with and without PCS. (**A**) Absolute neopterin values for asymptomatic (group 1) and symptomatic COVID-19 courses without PCS (group 2), as well as with PCS (groups 3, 4 and 5) at the time of the seropositive blood donation (0 M). Neopterin levels higher than 11 nmol/L (dashed line) are considered to be enhanced. (**B**) Relative change of neopterin levels in comparison to pre-pandemic blood donations in 2019

**Fig. 5:**
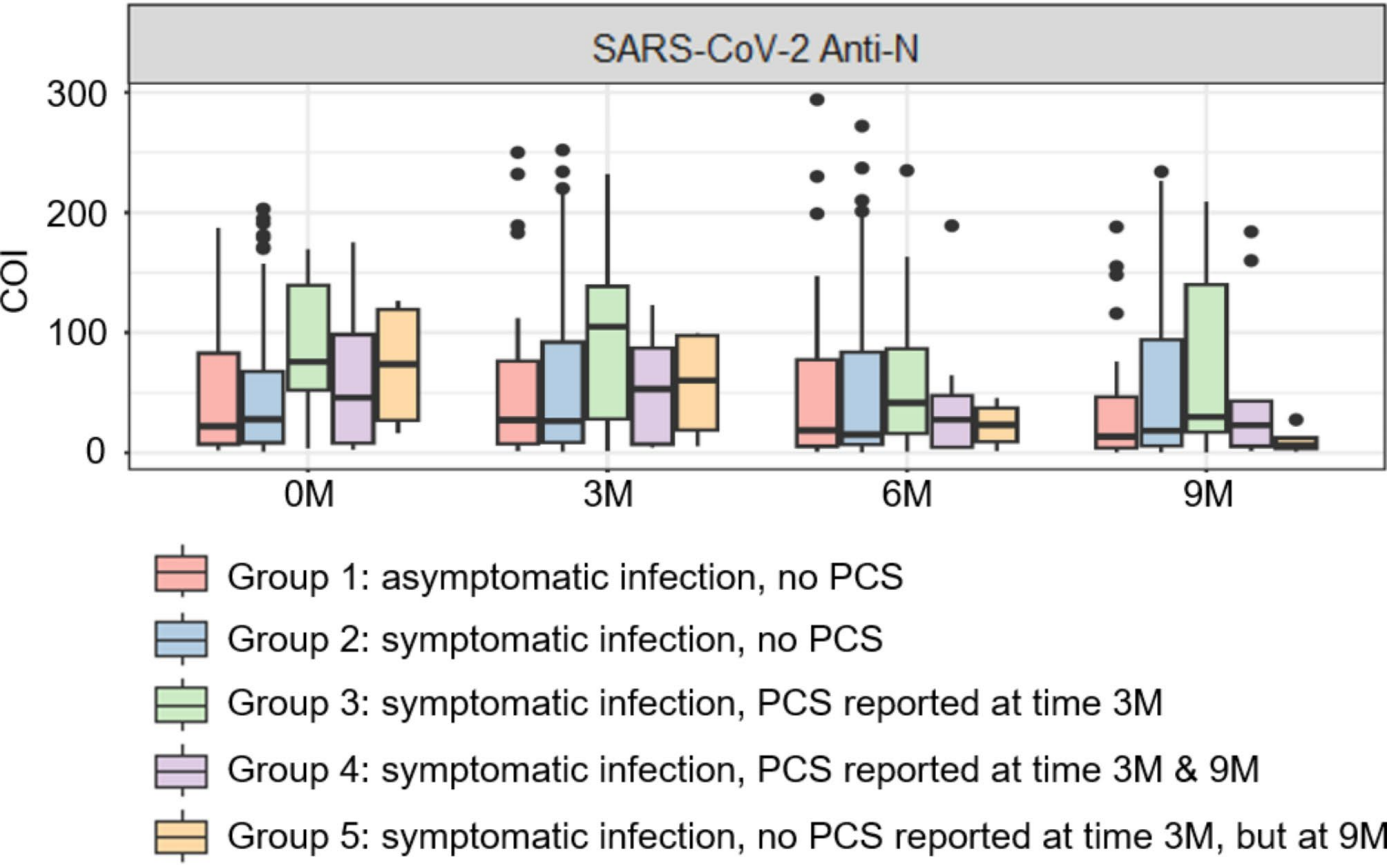
Developmental course of SARS-CoV-2 total anti-N antibodies spanning the first year after an infection. Data are shown as cut-off indices (COI) of SARS-CoV-2 anti-N total antibodies. 0 M = seropositive blood donation; 3, 6 and 9 M = 3, 6 and 9 months after the seropositive blood donation

**Fig. 6:**
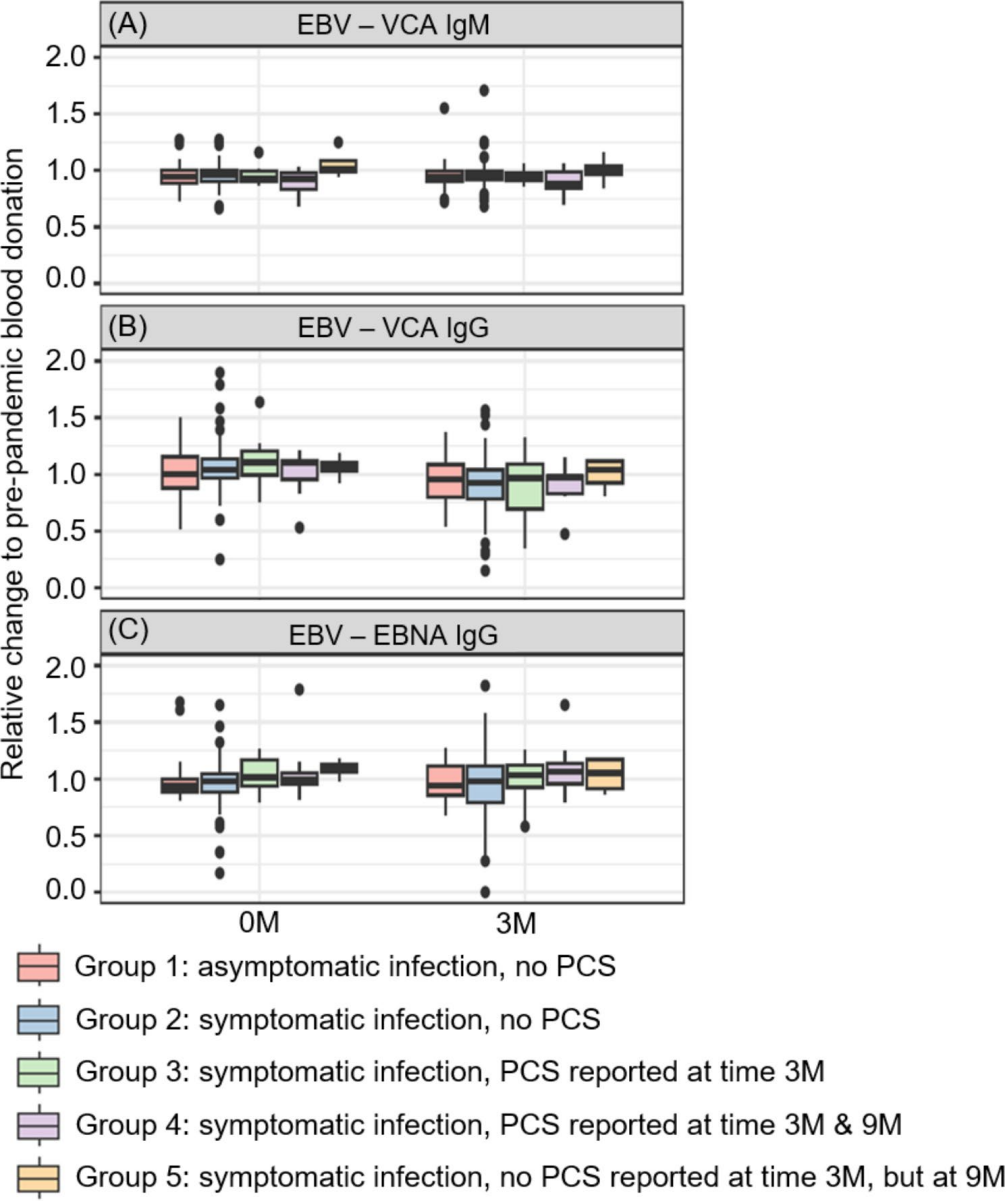
Levels of EBV-specific antibodies after a SARS-CoV-2 infection. (**A**) VCA IgM, (**B**) VCA IgG and (**C**) EBNA IgG antibody levels at the time of the SARS-CoV-2 seropositive blood donation (0 M) and 3 months thereafter (3 M) depicted as relative change to a pre-pandemic blood donation in the year 2019 of each participant. To obtain better descriptiveness of the figure shown, data with > 2.0 relative change of 21 individuals are not depicted in this plot

Figures 1, 2 and 3 were created with Microsoft Power Point 2016. Boxplots shown in Figs. 4, 5 and 6 as well as line plots in supplementary figure S1 were done by using the statistical software package R [31].

## Results

### 18% of SARS-CoV-2 Infections result in PCS with symptoms lasting for up to one year

For this study, 400 blood donors screened positive for SARS-CoV-2 anti-N total antibodies were included. This type of antibody is produced after a SARS-CoV-2 infection only, but not after vaccination, thus enabling to discriminate between infection-acquired and vaccination-acquired antibody response. All participants experienced either an asymptomatic or rather mild symptomatic COVID-19 disease course without hospitalisation. As shown in Figs. 2 and 351 participants answered the online surveys 3 and 9 months after the seropositive blood donation. As we intended to investigate a putative reactivation of EBV due to the SARS-CoV-2 infection, we further only included participants with available back-up serum and plasma samples from the pre-pandemic period in 2019 (n = 163) that were screened positive for long lasting EBV antibodies (VCA and EBNA IgG). Twelve of these 163 participants reported known pre-existing conditions including allergic asthma, hypothyroidism, hypertension and orthopaedic issues such as disc prolapse. Overall, the included 163 participants represent a healthy subgroup of an adult European population.

Using surveys that included questions about the course of the infection and putative long-lasting symptoms, we grouped the participants into the following 5 subgroups (Fig. 2): Group 1 reported an asymptomatic infection and no symptoms related to the infection that occurred at any later point in time (n = 26, 16%). The majority of participants were assigned to group 2, reporting a symptomatic SARS-CoV-2 infection with no persisting symptoms (n = 108, 66%). Members of group 3 reported a symptomatic SARS-CoV-2 infection with persisting symptoms 3 months after the initial seropositive blood donation (n = 15, 9%) which corresponds to a period of about 5–6 months after the infection [25]. Group 4 reported a symptomatic infection with persisting symptoms lasting up to 9 months, spanning about one year after an infection with SARS-CoV-2 (n = 10, 6%). Furthermore, 4 individuals (3%) with a symptomatic infection did not state persisting symptoms 3 months after the seropositive blood donation but reported symptoms assigned to the former SARS-CoV-2 infection after 9 months (group 5). In summary, 18% of all SARS-CoV-2 infections resulted in PCS with symptoms lasting for up to one year.

### Individuals with PCS are more likely to exhibit certain symptoms during COVID-19 disease

We examined which symptoms had occurred during a symptomatic disease course and compared individuals that did not report PCS (group 2, n = 108) with individuals reporting PCS (groups 3, 4 and 5, total n = 29). As shown in Table 1, we observed that individuals with PCS showed higher rates of symptoms during an ongoing infection such as cough (48% versus 41%), shortness of breath (38% versus 14%), headache (69% versus 44%) and body ache (66% versus 45%), hyposmia (55% versus 33%), dysgeusia (59% versus 33%) and GI-tract issues (14% versus 6%). In contrast, individuals with PCS reported less frequently a sore throat compared to individuals without PCS (17% versus 22%). Individuals with and without PCS reported comparable rates of fever (41% versus 39%) during the COVID-19 disease course. Furthermore, individuals with PCS reported more frequently to have experienced other symptoms including fatigue, nasal congestion, chills, weight reduction and pain of chest, bones and back (41% versus 23%). However, Fisher’s exact test did not reveal significant differences regarding the exhibition of particular symptoms between group 2 and the combination of groups 3, 4 and 5 (*p* > 0.05 for each symptom).

**Table 1:** Symptoms of individuals with and without PCS during COVID-19 disease course. Data are presented as number of individuals reporting a specific symptom and as percentages of the particular group. Other symptoms include fatigue, nasal congestion, chills, weight reduction and pain of chest, bones and back

	Group 2: Symptomatic infection, no PCS		Group 3: Symptomatic infection, PCS 3 M after initial BD		Group 4: Symptomatic infection, PCS 9 M after initial BD		Group 5: Symptomatic infection, no PCS after 3 M, but PCS 9 M after initial BD		Combination of PCS groups 3, 4 & 5	
	*Number of individuals*	*in %*	*Number of individuals*	*in %*	*Number of individuals*	*in %*	*Number of individuals*	*in %*	*Number of individuals*	*in %*
*Cough*	43	41	8	53	5	50	1	25	14	48
*Shortness of breath*	15	14	6	40	4	40	1	25	11	38
*Sore throat*	24	22	2	13	2	20	1	25	5	17
*Headache*	48	44	10	67	7	70	3	75	20	69
*Body ache*	49	45	12	80	5	50	2	50	19	66
*Hyposmia*	36	33	8	53	5	50	3	75	16	55
*Dysgeusia*	36	33	8	53	6	60	3	75	17	59
*GI-tract issues*	6	6	2	13	1	10	1	25	4	14
*Fever*	42	39	6	40	3	30	3	75	12	41
*Other symptoms*	25	23	8	53	4	40	0	0	12	41

### Fatigue, an altered sense of taste and shortness of breath are the most common persisting symptoms of PCS

As a next step, we asked participants with PCS about their long-lasting symptoms. Independent of the group assignment (group 3, 4 or 5), individuals described fatigue, an altered sense of taste (including hyposmia and dysgeusia) and shortness of breath as the three most common PCS symptoms (Fig. 3). Further symptoms reported cover a wide spectrum of health impairment and range from nasal congestion, cough, chest pain, body aches, headache, brain fog to elevated blood pressure and sleep disorders (Fig. 3).

### Low level of pro-inflammatory, antiviral immune response in individuals with and without PCS

Many Austrian blood collection centres routinely screen for neopterin, which is produced by activated monocytes, macrophages and dendritic cells upon stimulation by interferon gamma, which is generated by T-lymphocytes. Thus, neopterin is considered as a marker for an immune response dominated by activated T-helper cells. It was demonstrated to be elevated during the acute phase of numerous viral infections, including infections with e.g. hepatitis viruses, EBV, cytomegalovirus, measles, mumps and influenza viruses [23, 32]. Neopterin was also shown to be a valuable tool for the prediction of the COVID-19 disease course with elevated levels directly correlating with disease severity [33, 34]. Therefore, we questioned whether individuals with PCS may show enhanced levels of neopterin, indicating pro-inflammatory, active antiviral immune responses. As shown in Fig. 4A, at the time of the seropositive blood donation (~ 45–60 days after the acute phase of the infection) no elevated levels of neopterin (> 11 nmol/L) for any of the study groups was found. Furthermore, no significant difference between individuals with and without PCS was detected (*p* > 0.05) (Fig. 4A). We also compared the level of neopterin of each participant to the pre-pandemic blood donation of the same individual with no significant or visual differences in relative changes between the groups being observed (*p* > 0.05) (Fig. 4B).

### Comparable decline of SARS-CoV-2 anti-N antibodies in individuals with and without PCS

In order to analyse the specific antibody response in our study groups, the developmental course of SARS-CoV-2 anti-N total antibodies was examined. Our data revealed comparable levels of anti-N total antibodies for asymptomatic and symptomatic disease courses without PCS in the first year after an infection (Fig. 5). Individuals with symptomatic disease course followed by PCS tended to show higher anti-N total antibody levels 3 months after the initial seropositive blood donation, corresponding to 5–6 months post infection. However, a nonparametric ANOVA test showed that there is no significant difference between the groups. This trend was not observed for later points in time. In addition, no significant differences were found when combining groups 3, 4, 5 (n = 29) in comparison to group 1 (n = 26) and group 2 (n = 108), with *p* > 0.05 for each point in time and for the time-group interaction. Our data also reveal decreasing levels of SARS-CoV-2 total anti-N antibodies over time for all groups. The vast majority of participants assigned to PCS groups 3, 4 and 5 revealed constantly declining levels of total anti-N antibodies, corroborating the reports regarding reinfection, which was denied by all. However, one person of group 4 showed increased levels of anti-N antibodies (> 3-fold increase of COI value) after 9 months, although decreasing antibody levels at the times 3 and 6 months were detected. This indicates that putatively a reinfection had occurred that probably was unnoticed, which may explain the occurrence of SARS-CoV-2 related symptoms.

### EBV reactivation was not detected in individuals with PCS

Two studies reported that a SARS-CoV-2-associated reactivation of EBV in EBV-seropositive individuals might be causative for PCS [21, 22]. Applying qPCR, we examined whether EBV DNA could be detected in individuals reporting PCS (n = 29), which is considered as sign for EBV primary infection or reactivation [27, 28]. For each participant, we analysed the plasma samples from the pre-pandemic era, the initial blood donation and 3, 6 and 9 months afterwards. We could not detect EBV DNA for any participant reporting PCS at any point in time.

For each participant, we furthermore examined the levels of EBV-specific antibodies in a pre-pandemic blood donation from 2019, the seropositive blood donation and 3 months thereafter. We first examined the levels of EBV VCA IgM, which is together with EBV early antigen-diffuse (EA-D) IgG a transient antibody produced at an early stage of infection and thus frequently used to identify an EBV acute infection or reactivation [26, 27, 35]. As shown in Fig. 6A, we did not find enhanced levels of EBV VCA IgM in the groups reporting PCS. Furthermore, no significant differences between all groups in their relative change compared to the pre-pandemic blood donation were observed (*p* > 0.05). Since the VCA IgM antibody production is transient, we also examined the levels of long-lasting EBV antibodies VCA IgG and EBNA IgG, which are reported to show altered levels in the case of EBV reactivation [26, 27]. No significant differences were detected between the groups for the points in time investigated (*p* > 0.05). Moreover, no difference could be detected in the progression of the different antibody levels with respect to their relative values to the pre-pandemic donations (*p* > 0.05) (Fig. 6B and **C**).

Furthermore, we evaluated also a putative EBV antibody level change longitudinally for each of the 163 participants. As shown in Supplementary Figure S1, the majority of individuals categorized in groups 1 and 2 revealed comparable EBV IgM antibody levels over time. Only two individuals of these non-PCS groups showed an increase of more than 50% for IgM at time point three points after the blood donation. In contrast, no increase was observed over time for PCS-groups 3, 4 or 5. While the majority of participants also showed comparable levels of VCA and EBNA IgGs over time, some individuals showed an antibody level increase of more than 50%. However, as individuals of groups without PCS (groups 1 and 2) as well as participants of groups with PCS (groups 3, 4 and 5) revealed such an increase, this observation cannot be regarded as indication for EBV reactivation in individuals with PCS.

## Discussion

There are numerous causative mechanisms suggested to be responsible for the development of PCS, including the persistence of SARS-CoV-2 in different organs, presence or reactivation of other viruses such as EBV, activated autoimmunity and antiviral immune responses [12, 15–17]. Recent studies also suppose that SARS-CoV-2, similar to EBV, could modulate mitochondrial function, leading to increased cellular ageing, which might contribute to long-lasting symptoms [36]. However, data are still scarce and it remains elusive whether there is a single pivotal factor inducing PCS or a complex interplay of several factors including also aspects such as age, gender, genetic predispositions, comorbidities and general health status of the individual affected.

In this study, we focused on blood donors as representatives of a supposedly healthy, immunocompetent subgroup of an adult European population. While 16% of the included 163 individuals went through an asymptomatic disease course and had no PCS at a later point in time, 84% showed a rather mild course of COVID-19 with no hospitalisation. Even though the general health status of our study participants allowed a regular voluntary blood donation after the SARS-CoV-2 infection, 18% reported to suffer from PCS with symptoms lasting for up to one year after the infection. Thus, our data point out to a high incidence of sustained health impairment following SARS-CoV-2 infection in a per se healthy subgroup of the adult population, with approximately one in five individuals being affected.

We further found that individuals with PCS symptoms tend to have a higher probability to experience COVID-19 specific symptoms during the course of the disease such as head and body ache, cough, shortness of breath, GI-tract issues, hyposmia and dysgeusia. However, this observation is not statistically significant, as demonstrated by Fisher’s exact test. Furthermore, the course of COVID-19 was comparable to the one experienced by individuals without PCS, as no hospitalisation or other extensive medical care were required during the acute phase of the illness. The three most frequently reported long-lasting symptoms of PCS were independently of the PCS group assignment fatigue, an altered sense of taste (including hyposmia and/or dysgeusia) and shortness of breath. This is in line with the findings of other studies, which report these PCS symptoms to be among the most frequently reported ones [3, 11].

Our results showed that neopterin, a marker for an immune response dominated by activated T-helper cells upon stimulation (e.g. by viral infection), is not elevated in individuals reporting PCS. Furthermore, neopterin levels were not increased when compared to levels from the pre-pandemic period of the same individual. This lack of increase in neopterin levels in PCS blood donors suggests that an activation of macrophages and other immune cells, which are important to fight viral infections, does not take place at the time investigated. Thus, as neopterin was shown to be elevated during various viral infections including SARS-CoV-2 and EBV [23, 34], our data suggest that the acute phase of the viral infection seems to be successfully overcome in these individuals.

Regarding the specific immune response to SARS-CoV-2, our data point towards a similar response with respect to specific anti-SARS-CoV-2 antibody formation when comparing disease courses of COVID-19 with and without PCS. We found detectable but declining levels of specific anti-N antibodies in all groups within the first year after an infection, which is in line with our previous findings [25] and others [37]. Therefore, our data rather indicate a successful SARS-CoV-2 clearance than a viral persistence in individuals with PCS.

Some studies have indicated that in the course of a SARS-CoV-2 infection a reactivation of EBV may occur and that this viral reactivation directly correlates with severe COVID-19 disease courses [19, 20]. Furthermore, Gold et al. suggested that a higher frequency of EBV EA IgG antibodies, which was found in their patient cohort with PCS, could possibly point towards an EBV reactivation in such patients [21]. Another recently published study reveals that chronic viral coinfections including EBV, cytomegalovirus (CMV) and human immunodeficiency virus (HIV) may differentially affect the probability to develop PCS [38]. However, our data do not indicate a reactivation of EBV in healthy, immunocompetent individuals reporting PCS. qPCR screening of all individuals reporting PCS showed no detectable levels of EBV DNA at any point in time investigated, spanning the time from a blood donation during the pre-pandemic period in 2019 to one year after the SARS-CoV-2 infection.

The panel of EBV antibodies applied in this study (EBV VCA IgM, VCA IgG and EBNA IgG) is usually used to distinguish acute and past infections in immunocompetent individuals [27, 28, 39]. Comparing pre-pandemic and pandemic serum samples in the course of six months after a SARS-CoV-2 infection, we did not detect elevated or altered levels of EBV VCA IgM, VCA IgG or EBNA IgG antibodies neither did we detect any differences between individuals with and without PCS. Even though our data showed elevated levels of VCA and EBNA IgG antibodies for a few individuals compared to pre-pandemic times, this observation cannot be regarded as indication of EBV reactivation as members of PCS and non-PCS groups revealed such an increase. Thus, the longitudinal evaluation of EBV antibody levels showed no specific increase in individuals with PCS over time. Therefore, our results obtained from molecular biological and serological screenings suggest that EBV is not reactivated in healthy, immunocompetent individuals without known comorbidities who report PCS after a SARS-CoV-2 infection. These different results may be explained by rather heterogeneous study groups examined by Gold et al. and Peluso et al., which consisted of individuals with asymptomatic, mild and severe COVID-19 disease courses up to hospitalisation, but also persons with known comorbidities such as HIV-infection. In contrast, our data set includes only healthy and thus presumably immunocompetent adults.

The questionnaires used in this study were designed to cover different aspects of the SARS-CoV-2 infection and resulting COVID-19 among a rather healthy subgroup of the adult population, not solely to detect or document PCS. This approach is comparable to other studies [40, 41]. In the course of our online survey, participants were asked to name persisting symptoms they personally attributed to the SARS-CoV-2 infection, without using the terms “long-COVID” or “PCS” in the questionnaires.

Our study has some limitations. Regarding demographics, it should be noted that young individuals (< 18 years) and individuals older than 70 years were not included, as these age groups are not admitted to regular blood donation. Furthermore, as mentioned before, our findings might not be translated to individuals with certain comorbidities and severe disease courses. A certain bias due to self-reported PCS symptoms could apply. However, participants included in our study were selected after seropositive screening of total anti-N antibodies, assuring a previous SARS-CoV-2 infection. In addition, participants were aware that data regarding the general experience with COVID-19 disease were investigated in the course of the study, not necessarily PCS alone, thus limiting a self-selection bias to a certain degree. Furthermore, the exact time of infection could not be determined for all participants, as 16% experienced an asymptomatic disease course and some others could not give detailed information on the time of infection retrospectively. Strengths of our study are the rather homogenous study population of healthy, immunocompetent adults, and the pre-illness baseline data regarding known comorbidities. Furthermore, our study design includes pre-pandemic samples and samples collected at regular pre-defined points in time after a SARS-CoV-2 infection. This allows a direct comparison of neopterin levels, EBV DNA and EBV antibody levels from pre-pandemic times and different points in time after a SARS-CoV-2 infection, thus enabling a longitudinal evaluation of level changes for each participant.

To date we are not able to provide an explanation for PCS symptoms in our study cohort, as also reinfections with SARS-CoV-2 at a later point in time could not be detected for the vast majority of individuals with PCS when examining the developmental course of total anti-N antibody levels. However, a putative explanation could be a kind of over-reporting effect, in which certain symptoms are perceived with higher sensitivity, such as headache or fatigue, and are attributed to PCS due to ubiquitous media coverage. Nevertheless, we cannot exclude other factors (e.g., activated autoimmunity, altered mitochondrial functions or local viral persistence) that may cause PCS. Further investigations are clearly required to clarify the causative mechanisms of PCS in individuals with asymptomatic or mild COVID-19 disease course and to apply successful therapeutic interventions.

## Conclusions

In conclusion, our study reveals that PCS in per se healthy adults with no known comorbidities may not be explained by a reactivation of EBV as shown by screening for EBV DNA and specific antibodies. Furthermore, our data do not indicate a persisting pro-inflammatory, antiviral immune response or a specific SARS-CoV-2 antibody response, indicating that the SARS-CoV-2 infection as such seems to be successfully overcome in healthy adults approximately 45–60 days after the acute phase of the infection. Further examination is required to identify the cause of PCS in individuals with asymptomatic or mild COVID-19 disease course, which causes high social and economic burden for the individual affected and the society.

### Electronic supplementary material

Below is the link to the electronic supplementary material.

Supplementary Material 1

## Data Availability

The datasets generated and analysed during the current study are not publicly available due to data protection, but are available in an anonymized manner from the corresponding author on reasonable request.
