# Supplementary figures and images for: Epstein-Barr virus reactivation is not causative for post-COVID-19-syndrome in individuals with asymptomatic or mild SARS-CoV-2 disease course
Source: BMC Infect Dis. 2023 Nov 15;23:800. doi: 10.1186/s12879-023-08820-w (PMC10652630; doi:10.1186/s12879-023-08820-w)

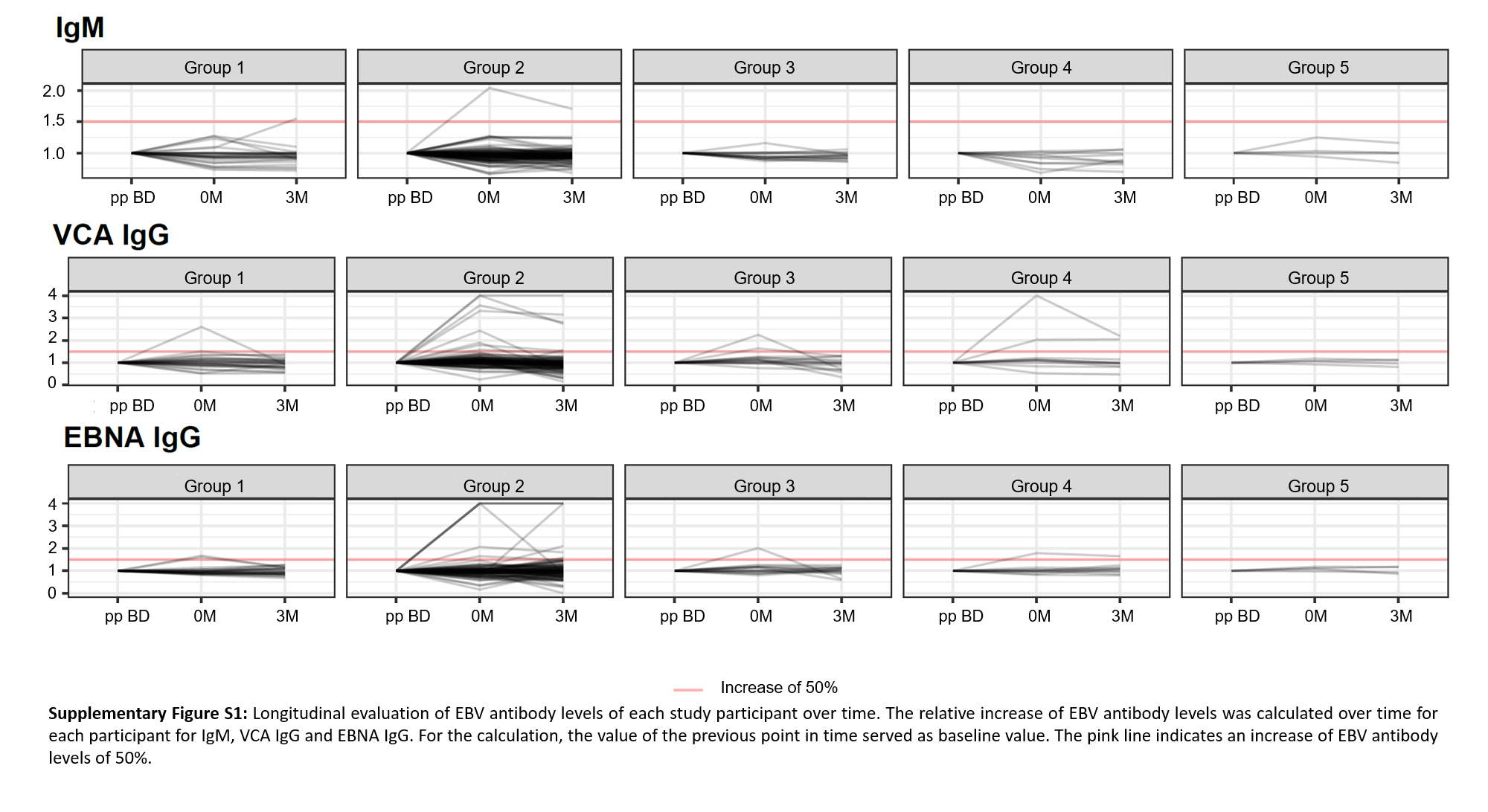

Supplement: Supplementary file 1 — Supplementary Material 1 [file 12879_2023_8820_MOESM1_ESM.png]
